# Fruit and Vegetable Intake Assessed by Food Frequency Questionnaire and Plasma Carotenoids: A Validation Study in Adults

**DOI:** 10.3390/nu7053240

**Published:** 2015-05-06

**Authors:** Tracy L. Burrows, Melinda J. Hutchesson, Megan E. Rollo, May M. Boggess, Maya Guest, Clare E. Collins

**Affiliations:** 1 School of Health Sciences, Faculty of Health and Medicine, Priority Research Centre for Physical Activity and Nutrition, University of Newcastle, Callaghan, NSW 2308, Australia; E-Mails: tracy.burrows@newcastle.edu.au (T.L.B.); melinda.hutchesson@newcastle.edu.au (M.J.H.); megan.rollo@newcastle.edu.au (M.E.R.); mboggess@asu.edu (M.M.B.); maya.guest@newcastle.edu.au (M.G.); 2 School of Mathematical and Statistical Sciences, Arizona State University, Tempe, AZ 85287, USA

**Keywords:** diet assessment validation, food frequency questionnaire, fruit vegetable intake, plasma carotenoids

## Abstract

Dietary validation studies of self-reported fruit and vegetable intake should ideally include measurement of plasma biomarkers of intake. The aim was to conduct a validation study of self-reported fruit and vegetable intakes in adults, using the Australian Eating Survey (AES) food frequency questionnaire (FFQ), against a range of plasma carotenoids. Dietary intakes were assessed using the semi-quantitative 120 item AES FFQ. Fasting plasma carotenoids (α- and β-carotene, lutein/zeaxanthin, lycopene and cryptoxanthin) were assessed using high performance liquid chromatography in a sample of 38 adult volunteers (66% female). Significant positive correlations were found between FFQ and plasma carotenoids for α-carotene, β-carotene and lutein/zeaxanthin (52%, 47%, 26%, *p* < 0.001, 0.003, 0.041; respectively) and relationships between plasma carotenoids (except lycopene) and weight status metrics (BMI, waist circumference, fat mass) were negative and highly significant. The results of the current study demonstrate that carotenoid intakes as assessed by the AES FFQ are significantly related to plasma concentrations of α-carotene, β-carotene and lutein/zeaxanthin, the carotenoids commonly found in fruit and vegetables. Lower levels of all plasma carotenoids, except lycopene, were found in individuals with higher BMI. We conclude that the AES can be used to measure fruit and vegetable intakes with confidence.

## 1. Introduction

Regular consumption of fruit and vegetable intake in accordance with World Cancer Research Fund guidelines is associated with a reduced risk of some cancers and substantially lower risks of coronary heart disease [1,2], stroke [3,4] and possibly type 2 diabetes mellitus [5,6].

Plasma biomarkers can be used in studies validating dietary intake as independent proxy measures of intake [7] and to evaluate whether sources of random error are independent of errors associated with measurement by questionnaire and/or inaccuracies within nutrient databases [8]. Simultaneous measurement of plasma carotenoid concentrations have been reported in studies validating fruit and vegetable intakes [9,10] as carotenoids predominate in these foods [11]. It has been reported that a single carotenoid is not likely to be sufficient due to the diverse composition of plant foods [12]. Alpha-carotene, β-carotene, cryptoxanthin, lycopene and lutein are the carotenoids most commonly assessed in dietary validation studies [13].

Therefore, the aim of the current study was to compare fasting plasma carotenoid concentrations, as biomarkers of fruit and vegetables, with dietary carotenoids and intakes of fruit and vegetables, as assessed by the Australian Eating Survey (AES) food frequency questionnaire (FFQ) in a sample of adults.

## 2. Experimental Section

Data used in this analysis were obtained from a convenience sample of adults who had previously participated in a comparative validation study (The Family Diet Quality Study) [14] and who volunteered to give a blood sample for plasma carotenoid analysis. The methods have been published previously elsewhere [14]. Briefly, participants were recruited into the validation study through advertisements in newspapers, community notice boards and school newsletters in Newcastle, New South Wales, Australia. Eligibility included being an adult (>18 years), with no known medical conditions or taking medications that could influence body weight, (e.g., asthma, type 1 diabetes) and living full-time with at least one child aged 8–10 years, with data collected as part of a study of nutrition in families [14]. Demographic data including age, education, smoking status and self-rated health were measured. Anthropometric data, including height (cm), weight (kg) and body mass index (BMI) calculated as kg/m^2^, waist circumference (cm) and fat mass (kg) were measured by trained assessors with full details reported elsewhere [15]. A trained research assistant explained and administered the AES FFQ, which consisted of 120 items, reporting intake over the previous six months. This instrument has previously been used compared in adults to weighed food records without use of independent biomarkers [14].

Fruit and vegetables were reported as servings per day. An individual response for each food or food type was recorded with seven frequency options ranging from “never” up to “4 or more times per day” and for some beverages up to “7 or more glasses per day”. Nineteen FFQ items related directly to intake of vegetables. Vegetable types assessed were potatoes, pumpkin, sweet potato, cauliflower, green beans, spinach (*i.e*., Swiss chard), cabbage/brussels sprouts, peas, broccoli, carrots, zucchini, eggplant, summer squash, capsicum (*i.e*., red and green bell pepper), corn, mushrooms, tomatoes, lettuce, celery, cucumber, avocado, onion, spring onion, and leek. Eleven FFQ items related directly to the intake of fruit. Fruits assessed were: canned fruit, fruit salad, dried fruit, apple, pear, orange, mandarin, grapefruit, banana, peach, nectarine, plum or apricot, mango, paw-paw, pineapple, grapes, strawberries, blueberries and melon. The frequency categories for seasonal fruit for fruits such as peach and melon were calculated by adjusting for the number of months per year the fruit was available. Vitamin supplement use was assessed as “yes” or “no”.

Standard adult portion sizes were used for each food item and derived from the most current Australian National Nutrition Survey using unpublished data purchased from the Australian Bureau of Statistics [16]. Daily carotenoid intakes of α-carotene, β-carotene, lycopene, cryptoxanthin and combined lutein-zeaxanthin were estimated from FFQ fruit and vegetable responses using the carotenoid database from the US Department of Agriculture National Cancer Institute [17].

### 2.1. Biochemical Assay

Phlebotomists collected blood samples in EDTA-coated tubes after an overnight fast and samples were analysed at an accredited pathology service (National Association of Testing Authorities, Australia). Serum was separated from red blood cells by centrifugation and remaining samples were frozen within 2 h to −80 °C. Samples were thawed and high performance liquid chromatography (HPLC) methodology was used to determine β-carotene, lycopene, α-carotene, β-cryptoxanthin and lutein/zeaxanthin concentrations in serum. All extractions were carried out in a darkened laboratory under red light. In a 1:1 ratio, ethanol plus ethyl acetate containing internal standard (canthaxanthin) were added to the sample. The solution was vortexed, centrifuged (3000 g, 4 °C for 5 min) and the supernatant was collected. This process was repeated three times, adding ethyl acetate twice, then hexane to the pellet. Ultra-pure water was then added to pooled supernatant and the mixture was vortexed and centrifuged. The supernatant was decanted, the solvents evaporated with nitrogen and the sample reconstituted in dichloromethane:methanol (1:2 v/v). Chromatography was performed on a Hypersil ODS column (100 mm × 2.1 m × 5 μm) with a flow rate of 0.3 mL/min. Carotenoids were analysed using a mobile phase of acetonitrile: dichloromethane: methanol (containing 0.05% ammonium acetate) (85:10:5 v/v) and a diode array detector (470 nm) [18].

### 2.2. Ethics

The study protocol was approved by the University of Newcastle Human Research Ethics Committee (Approval No. H-2010-1170).

### 2.3. Statistical Methods

Median, minimum and maximum values were reported for reported FFQ fruit and vegetable intakes, FFQ carotenoids and plasma carotenoid concentrations. BMI was significantly associated with plasma carotenoids, as expected [19,20], and thus was used to stratify descriptive statistics. Participants were categorized as either healthy weight (BMI < 25 kg/m^2^) or overweight (BMI ≥ 25 kg/m^2^). Univariate effects (Table 1 and Table 2) were assessed using Wilcoxon rank-sum tests. Comparisons of plasma carotenoids to FFQ carotenoids were made using Wilcoxon matched pairs signed-rank tests. Linear regression models were used to assess the relationship between plasma and FFQ carotenoid levels, whilst controlling for anthropometric variables (BMI, waist circumference and fat mass). Clustered, robust standard errors were used to account for the probable correlation of food intakes of participants in the same family [21]. The normality of the residuals from these models was assessed using the Shapiro-Wilk’s test. Statistical significance is determined at the 5% level. All statistical analysis was performed using Stata MP version 12 [22].

**Table 1:** Anthropometric summary for *n* = 38 participants from 26 families, by weight category.

	All		Healthy Weight (BMI < 25)		Overweight (BMI ≥ 25)		*p*
	*n* = 38		*n* = 20		*n* = 18		
Female	25 (66%)		14 (70%)		11 (61%)		0.73
Supplement use	20 (53%)		10 (50%)		10 (56%)		0.76
	Median	(Min–Max)	Median	(Min–Max)	Median	(Min–Max)	
Age (years)	43.3	(33.5–52.6)	42.9	(36.8–50.6)	44.9	(33.5–52.6)	0.64
Height (cm)	169.3	(151.4–188.0)	169.8	(161.6–184.5)	168.3	(151.4–188.0)	0.24
Weight (Kg)	68.8	(55.6–99.6)	64.4	(55.6–78.5)	79.4	(61.5–99.6)	<0.01
BMI (kg/m^2^)	24.4	(19.4–37.8)	22.5	(19.4–24.5)	27.9	(25.1–37.8)	<0.01
Waist (cm)	83.4	(67.7–111.4)	78.8	(67.7–91.4)	91.1	(76.9–111.4)	<0.01
Fat Mass (Kg)	21.3	(7.0–48.3)	14.6	(7.0–23.8)	24.5	(14.5–48.3)	<0.01
Fat Mass (%)	26.6	(11.2–50.9)	20.8	(11.2–35.3)	34.3	(17.8–50.9)	<0.01
Fat Free Mass (Kg)	48.1	(38.9–77.4)	48.1	(40.6–63.4)	48.5	(38.9–77.4)	0.70
Fat Free Mass (%)	73.4	(49.1–88.8)	79.2	(64.7–88.8)	65.7	(49.1–82.2)	<0.01

*p* Value indicates differences between weight groups.

**Table 2:** Summary statistics (median, minimum and maximum) for plasma carotenoids, food frequency questionnaire (FFQ) carotenoids and fruit and vegetable intake, by weight category.

	All		Healthy Weight (BMI < 25)		Overweight (BMI ≥ 25)		*p*
	*n* = 38		*n* = 20		*n* = 18		
	Median	(Min–Max)	Median	(Min–Max)	Median	(Min–Max)	
Plasma Carotenoid (µg/dL)							
α-carotene	6.40	(0.80–29.30)	7.35	(1.90–29.30)	3.85	(0.80–28.40)	0.05
β-carotene	40.65	(3.50–176.80)	46.4	(7.30–162.40)	25.4	(3.50–176.80)	0.01
Lycopene	40.85	(7.20–114.30)	38.7	(7.20–114.30)	43.3	(13.40–94.60)	0.64
Lutein-zeaxanthin	21.05	(7.50–64.60)	24.95	(9.40–64.60)	16.3	(7.50–38.40)	0.05
Cryptoxanthin	7.60	(1.70–18.80)	8.95	(1.70–16.50)	5.25	(2.70–18.80)	0.06
	Median	(Min–Max)	Median	(Min–Max)	Median	(Min–Max)	
FFQ Carotenoid (μg/day)							
α-carotene	12.78	(3.67–74.16)	12.8	(3.67–74.16)	12.15	(4.30–26.89)	0.88
β-carotene	57.72	(11.98–191.3)	57.65	(11.98–191.33)	59.84	(26.09–98.53)	0.98
Lycopene	95.27	(34.09–194.3)	89.15	(34.09–194.28)	96.11	(39.27–176.69)	0.54
Lutein-zeaxanthin	29.35	(6.49–72.9)	30.78	(6.49–72.89)	29.11	(12.35–52.96)	0.27
Cryptoxanthin	3.56	(0.45–9.18)	3.56	(0.45–9.18)	3.42	(1.00–7.59)	1
FFQ Vegetables (serves/day)							
All	4.26	(1.07–9.07)	4.43	(1.57–9.07)	3.94	(1.07–6.71)	0.24
FFQ Fruit (serves/day)							
All	2.57	(0.20–4.71)	2.57	(0.20–4.01)	2.55	(0.30–4.71)	0.77

*p* Value indicates differences between weight groups.

## 3. Results

### 3.1. Descriptive Statistics

A total of 38 participants (*n* = 25, 66% female) from 26 families completed the FFQ and provided a blood sample for plasma carotenoid measurement. Twenty participants (53%) were healthy weight and 18 (47%) were classified as overweight. Twenty nine participants reported having no health conditions; health conditions reported by 9 participants were back pain (*n =* 3), asthma (*n =* 3), depression (*n =* 2), arthritis (*n =* 2) and one each of anxiety, high blood pressure, low blood pressure, high cholesterol, heart murmur, stent due to cardio-vascular disease, and Crohn’s disease. The majority had completed a high school or trade education (*n =* 34, 89%). Just over half (*n =* 20, 53%) reported using vitamin supplements. One participant was a current smoker and two were previous smokers. Table 1 reports anthropometric measures for the total sample and by BMI status category (over or under 25), with no significant differences between groups for age, sex, height and fat-free mass (kg).

Mean participant macronutrient intakes indicated that 18% of energy intake was derived from protein, 47% carbohydrate, 31% fat and 12% saturated fat and this did not differ by BMI status category. Mean fruit intake was 2.5 servings/day and 4.4 servings/day for vegetables with no difference by BMI status category. Mean consumption of orange and yellow vegetables (carrot, pumpkin, sweet potato and corn) was 1.2 serves/day, red vegetables (tomato) 0.5 serves/day and green vegetables (spinach, cabbage and brussel sprouts) <0.5 serves/day with no difference by BMI status category.

Summary statistics for plasma and FFQ carotenoids and intakes of selected FFQ fruit and vegetables by BMI status category are reported in Table 2. While there were no significant differences in dietary carotenoid intakes by BMI status category (all *p*-values >0.2), there were significantly lower concentrations in plasma β-carotene carotenoids for overweight compared to healthy weight (*p =* 0.01), and marginally lower plasma concentrations for α-carotene, lutein-zeaxanthin and cryptoxanthin (*p =* 0.05, 0.05 and 0.06 respectively).

### 3.2. Linear Regression Modelling

Table 3 reports the correlations and partial correlations from multivariate regression models with plasma carotenoids as the response and anthropometrics, FFQ carotenoids and FFQ fruit and vegetable intakes as explanatory variables that were significant at the 5% level.

**Table 3:** Correlations between Food Frequency Questionnaire carotenoid intake and plasma carotenoid concentrations from multivariable linear regression modelling of plasma carotenoids, controlling for Body Mass Index and fat mass, significant at the 5% level.

Anthropometric	*p*	FFQ Intake	*p*	Model R-squared	FFQ—Plasma Correlation	Correlation 95% CI
Plasma α-carotene						
		α-carotene	<0.001	0.26	0.52	0.35, 0.69
BMI	0.004	α-carotene	<0.001	0.34	0.49	0.33, 0.64
BMI	0.001			0.11		
Plasma β-carotene						
		β-carotene	0.003	0.21	0.47	0.18, 0.75
		Veg Serves	0.007	0.17	0.42	0.12, 0.71
BMI	0.016	β-carotene	0.004	0.31	0.41	0.15, 0.68
Fat mass	0.013	Veg Serves	0.013	0.25	0.34	0.08, 0.61
BMI	0.004			0.14		
Fat mass	0.003			0.14		
Plasma Lycopene						
		Lycopene	0.756	0.00		
Plasma Lutein/zeaxanthin						
		Lutein/zeax	0.041	0.09	0.26	0.01, 0.51
BMI	<0.001	Lutein/zeax	0.095	0.20		
BMI	<0.001			0.14		
Plasma Cryptoxanthin						
		Cryptoxant	0.236	0.08		
Fat mass	0.005	Supplements	0.003	0.35		
Fat mass	<0.001			0.22		

**Figure 1:**
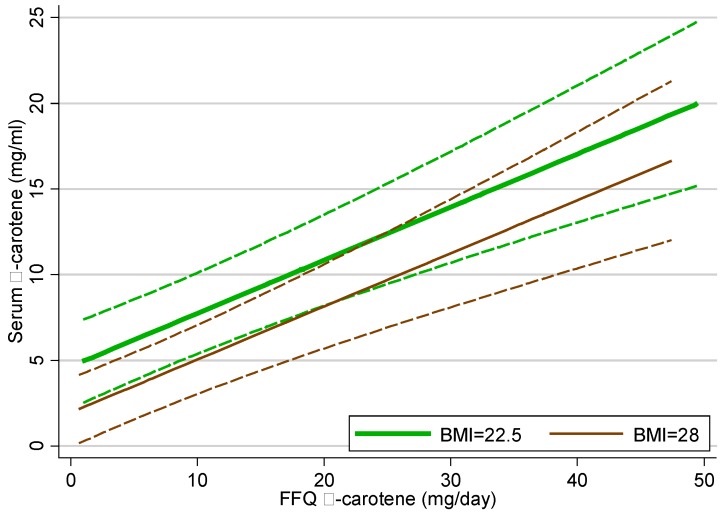
Estimated mean plasma α-carotene from multivariate linear regression model on FFQ α-carotene by BMI status, with 95% confidence interval. BMI = 22.5 and BMI = 28 were selected as these were the median BMI for the groups used in descriptive statistics shown Table 2.

The correlation between plasma α-carotene concentration and FFQ dietary α-carotene intake was 0.52. FFQ dietary α-carotene was significantly (*p* < 0.001) related to plasma α-carotene concentration, whilst controlling for BMI (*p =* 0.007). For purposes of demonstration, Figure 1 shows estimated mean plasma α-carotene concentration, for an individual with BMI = 22.5 or 28, by α-carotene intake as reported in the FFQ. The strong positive slopes seen in Figure 1 demonstrate that plasma α-carotene increases in line with FFQ α-carotene intakes; more precisely for an increase in FFQ α-carotene intake of 1 mg/day there was a corresponding plasma α-carotene concentration increase of 0.310 mg/mL. The interaction term in the model was not significant, indicating that this relationship does not change with BMI. The model R-squared was 0.34, which was considerably higher than the univariate model containing BMI alone (*R*-squared = 0.11), or that with FFQ α-carotene alone (*R*-squared = 0.26), demonstrating that FFQ α-carotene dietary intake has substantial predictive power beyond that of BMI alone.

**Figure 2:**
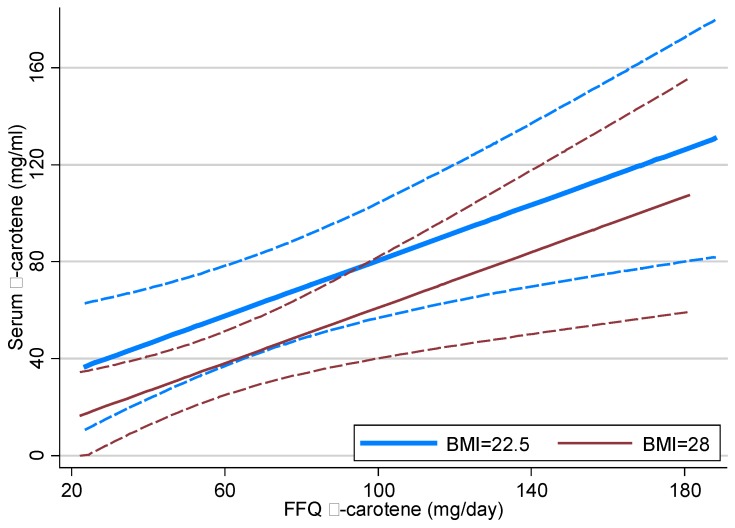
Estimate mean plasma β-carotene from multivariate linear regression model on FFQ β-carotene by BMI status, with 95% confidence interval.

The correlation between plasma β-carotene concentration and FFQ dietary β-carotene intake was 0.47, and between plasma β-carotene concentration and FFQ dietary vegetable serves per day was 0.42. Plasma β-carotene was significantly related to FFQ β-carotene and BMI. The *p*-values from the model (*p =* 0.001, *p =* 0.018, respectively) indicated significant relationships between plasma β-carotene and FFQ β-carotene. The R-squared of this model was substantially greater (*R*-squared = 0.31) than for the model that included BMI alone (*R*-squared = 0.14). Figure 2 displays estimated mean plasma and FFQ β-carotene by BMI. More than any of the other plasma carotenoids, plasma β-carotene was significantly related to FFQ total vegetable intake, expressed as number of serves per day (*p =* 0.013), while controlling for fat mass, with *R*-squared equal to 0.25.

The correlation between plasma lutein-zeaxanthin concentration and FFQ dietary lutein-zeaxanthin intake was 0.29. Plasma lutein-zeaxanthin was significantly related to FFQ dietary lutein-zeaxanthin (*p =* 0.041), but the significance level was reduced when BMI was included in the model (*p =* 0.095).

In contrast to the other carotenoids, plasma concentrations of neither lycopene nor cryptoxanthin were significantly correlated with FFQ dietary intake (*p =* 0.756, 0.236, respectively). Lycopene was not significantly related to BMI, waist circumference or fat mass (*p =* 0.55, 0.34, 0.16, respectively).

Supplement use (*n =* 20 out of 38 participants) was significantly associated with plasma cryptoxanthin (*p =* 0.003), whilst controlling for fat mass (*p =* 0.005), with an *R*-squared of 0.35. There was a negative relationship between plasma cryptoxanthin and fat mass, with plasma concentrations decreasing in line with increasing fat mass, and that supplement users have higher plasma cryptoxanthin. Supplement use was not significantly associated to plasma concentrations for α-carotene, β-carotene, lycopene or lutein-zeaxanthin (*p =* 0.37, 0.48, 0.20, 0.81, respectively).

Both fat mass and waist circumference were significantly related to plasma carotenoids, although the relationship with waist circumference was less significant, and thus it was not reported. Fat mass significance was reported in this study only if it was substantially greater than that of BMI. No significant relationships were found between any plasma carotenoid and age or sex.

## 4. Discussion

The aim of the current study was to compare plasma carotenoid concentrations, as biomarkers of fruit and vegetable intake, to examine the relative validity of fruit and vegetable intake self-reported using a semi-quantitative FFQ in a sample of adults. Highly significant correlations were found between dietary intake and plasma concentrations for two of the five measured carotenoids, α- and β-carotene. These two are the most abundant carotenoids in the food supply and are primarily found in yellow and orange coloured fruits and vegetables. These foods were consumed commonly in this group, which is similar to another Australian report [19].

A dose-response relationship between food intake and appearance of carotenoids in plasma has been demonstrated by previous researchers in large prospective cohort studies in adults [23] providing support for their use as reliable biomarkers of intake. In the majority of previous studies, correlations between dietary carotenoid intake and plasma carotenoid concentrations have been variable, with correlations ranging from 0.2 to 0.7, and most studies showing statistical significance for at least one of the primary carotenoids measured [10,24,25]. The most commonly reported associations between diet and plasma carotenoids have been with the provitamin A compounds α- and β-carotene and this has been confirmed in the current study. This may be attributed to both these carotenoids, having a higher bioavailability than other fruits and vegetables, as it is not part of a protein complex as in the case when found in green leafy vegetables [26]. A lack of correlation between plasma lycopene and dietary intake of lycopene [9] and fruit and vegetable intake [27] has been previously reported and suggests that not all food high in lycopene were captured by the FFQ or that other variable confound the relationship.

Weight status [19], supplementation [28] and smoking status [29] have been previously shown to influence plasma carotenoids concentrations in humans, and thus need to be accounted for when estimating the relationship between plasma carotenoids and food intake. In the current sample one person was a smoker and two were previous smokers so no effect of smoking could reliably be estimated. The impact of supplement use assessed in the current study was found to only significantly influence plasma levels for cryptoxanthin. This may be because dietary sources high in cryptoxanthin such as orange rind and papaya are not as frequently consumed, and are often in much smaller concentrations compared with other carotenoids including α and β-carotene. Previous studies have found supplements to have a general influence across a range of carotenoids rather than a specific one such as only cryptoxanthin as in the current study, although differences between supplement and non-supplement users have mixed [9,30].

The relationships between carotenoid concentrations and BMI found in this study concur with other studies in adults and children that suggest there may be a physiological mechanisms operating. For example, as BMI increases circulating carotenoids reduce secondary to increased utilization [31], or there may be differences in absorption and metabolism secondary to higher weight status [19,23,32]

A limitation of the current study was the small sample size and thus a possibly small variation in intakes. Also, the relatively small number of foods in the nutritional database used, although this is comparable with other dietary validation studies in adults [33]. Carotenoid databases available currently for use in estimating dietary intakes of carotenoids are not comprehensive and although the USDA database used in this study [17] was updated in 2006 there were still only 40 of the 120 items in the AES FFQ that have been evaluated for carotenoid content. However this was superior to the current Australian database which is even more limited, with approximately only 25 of the foods from the AES FFQ having values estimated. This limitation is likely to have reduced the likelihood of detecting relationships between dietary intakes and plasma carotenoid concentrations as significant. While the US carotenoid data values may not completely reflect current Australian food values, at the time of analysis it was the most comprehensive database of fruit and vegetable intake internationally.

A strength of the current study is that is demonstrates the approach to using a plasma biomarker as an independent assessment of dietary intake, in this case plasma carotenoids, dietary carotenoids and dietary fruit and vegetable intake. This approach can be used to guide researchers in the design of other such studies.

## 5. Conclusions

In conclusion, the results of the current study demonstrate that carotenoid intakes, as assessed by the AES FFQ are significantly related to plasma carotenoid concentrations of α-carotene, β-carotene and lutein/zeaxanthin, the carotenoids commonly found in fruit and vegetables. Lower levels of all plasma carotenoids, except lycopene, were found in individuals with higher BMI. We conclude that the AES can be used to measure fruit and vegetable intakes with confidence.
